# A comprehensive Chinese experience against SARS-CoV-2 in ophthalmology

**DOI:** 10.1186/s40662-020-00187-2

**Published:** 2020-04-07

**Authors:** A-Yong Yu, Ruixue Tu, Xu Shao, Anpeng Pan, Kaijing Zhou, Jinhai Huang

**Affiliations:** Eye Hospital of Wenzhou Medical University, 270 Xueyuan West Road, Wenzhou, 325000 Zhejiang P. R. China

**Keywords:** Coronavirus, SARS-CoV-2, COVID-19, Ophthalmology, Ocular transmission

## Abstract

The 2019 novel coronavirus disease (COVID-19) has now swept through the continents and poses a global threat to public health. Several investigations have been conducted to identify whether COVID-19 can be transmitted through the ocular route, and the conclusion is that it is a potential route but remains uncertain. Due to the face-to-face communication with patients, frequent exposure to tears and ocular discharge, and the unavoidable use of equipment which requires close proximity, ophthalmologists carry a high risk of contracting severe acute respiratory syndrome coronavirus 2 (SARS-CoV-2). Based on 33 articles published by Chinese scholars, guidelines and clinical practice experience in domestic hospitals, we have summarized the Chinese experience through the lens of ophthalmology, hoping to make a contribution to protecting ophthalmologists and patients around the world.

## Background

Since the first pneumonia patient was identified around December 2019, in Wuhan, China, multiple human cases of severe acute respiratory syndrome coronavirus 2 (SARS-CoV-2) infection have been reported. The 2019 novel coronavirus disease (COVID-19) has now swept through the continents and poses a global threat to public health. Up till 12th March 2020, at least 80,980 cases in China and 43,538 cases beyond China were confirmed, covering 118 countries, areas or territories.

Many infections amongst medical staff have been reported, of whom three ophthalmologists from Wuhan Central Hospital died of COVID-19 due to occupational exposure, and Dr. Guangfa Wang, a pneumonia expert, was infected by SARS-CoV-2 through unprotected eye exposure. These events raise an alarm on the route of SARS-CoV-2 transmission. Faced with the possibility of ocular transmission, ophthalmologists are very likely to contract the infection. Drawing on the rich experience during the previous SARS outbreak, the Chinese government has promptly released various protection measures for ophthalmology, and recommended protection for the eyes, as well as mouth and nose, when caring for patients potentially infected with SARS-CoV-2. The American Academy of Ophthalmology recently published a similar recommendation for ophthalmologists from the Centers for Disease Control and Prevention (CDC). Based on the latest published literatures, guidelines and clinical practice experience in domestic hospitals, we have summarized the Chinese experience through the lens of ophthalmology, hoping to make a contribution to protecting ophthalmologists and patients around the world, and praying that the pandemic will be contained as soon as possible.

## Main text

### Articles on SARS-CoV-2 for ophthalmology from Chinese scholars

We searched MEDLINE, ScienceDirect, Embase, the Cochrane Library, WanFang Database, VIP Database, SinoMed, China National Knowledge Infrastructure (CNKI), the CDC for COVID-19 website (https://www.cdc.gov/coronavirus/2019-ncov/publications.htm), Chinese Scientific Research Academic Exchange Platform for COVID-19 (http://medjournals.cn/2019NCP/index.do), and relevant references for papers related to "ophthalmology and SARS-CoV-2/COVID-19"; published till 12^th^ March 2020. The search strategy was as follows: (SARS-CoV-2 or 2019-nCov or COVID-19 or NCP or coronavirus or "severe acute respiratory syndrome coronavirus 2" [Supplementary Concept] or "COVID-19" [Supplementary Concept]) and (ocular or eye or ophthalm* or ophthalmologist or tear or conjunctiv* or "Conjunctivitis"[Mesh] or "Conjunctivitis, Viral"[Mesh]).

We identified 33 articles in total published by Chinese scholars directly relevant to ophthalmology and SARS-CoV-2/COVID-19. Twenty-seven articles are published in Chinese journals, most articles are reviews, almost all regarding ophthalmic precautions and ocular surface transmission of SARS-CoV-2 infection (Table 1).

**Table 1:** A summary of published articles by Chinese scholars directly relevant to ophthalmology and SARS-CoV-2/COVID-19

Study type	Language	N	Date (yyyy.mm.dd)	City	Key Point
[1] Retrospective cohort study	English	67	2020.02.11	Wuhan	SARS-CoV-2 can be detected in the conjunctival sac of patients with COVID-19. Through clinical analysis, viral transmission via the conjunctival route was not supported by the data. Good clinical protection can effectively cut off the transmission path.
[2] Cross-sectional study	English	102	2020.02.26	Wuhan	The nosocomial infection of SARS-CoV-2 through the eyes after occupational exposure is a potential route. Protective goggles should be worn by all healthcare workers.
[3] Prospective case series study	English	30	2020.02.26	Hangzhou	SARS-CoV-2 may be detected in the tears and conjunctival secretions of COVID-19 patients with conjunctivitis.
[4] Editorial	English	–	2020.03.03	Hong Kong	As the novel corona-virus infection is still on the upward trend, it is of paramount importance to remain vigilant and start taking necessary measures to prevent its transmission.
[5] Editorial	English	–	2020.02.13	Beijing, Hong Kong, Guangzhou	Ophthalmologists should take particular care when examining patients, research studying whether COVID-19 can be found in tears and conjunctival scrapings would be valuable and inform ongoing disease-prevention strategies.
[6] Letter	English	–	2020.02.22	Changchun	SARS-CoV-2 transmission through the ocular surface must not be ignored.
[7] Review	Chinese	–	2020.02.22	Beijing	A COVID-19 patient with ocular syndrome was reported and treated combined with traditional Chinese medicine (TCM). The article discussed COVID-19 from the perspective of TCM.
[8] Review	Chinese	–	2020.02.23	Beijing	Research of beta coronavirus receptors on ocular surfaces found two host receptors, ACE2 and DPP4. Their expression in the cells of ocular surface may be an access route of corona virus in eye, which provides clues to elucidating the pathogenesis of corona virus in the eyeballs.
[9] Review	Chinese	–	2020.02.13	Wenzhou	This article preliminarily reviews several different aspects, including the characteristics of SARS-CoV-2, the anatomical connection between ocular surface and respiratory tract, previous work on the link between respiratory virus and ocular complication, and the data of ACE2 receptor expression and molecular detection of SARS virus in tear. Based on these reviews, intervention measures are advised for ophthalmic practitioner, whilst suggestions are indicated for further clinical and basic investigations in the future.
[10] Review	Chinese	–	2020.02.04	Wuhan	It is extremely important to disinfect ophthalmic examination instruments and protect ophthalmic medical care during the epidemic period to reduce cross-infection in clinical practice. Some suggestions against SARS-CoV-2 infection were offered in this article.
[11] Review	Chinese	–	2020.02.12	Multi-center	Ophthalmic experts, from Society of Public Health Ophthalmology, Chinese Preventive Medicine Association and Beijing Ophthalmological Society and Youth Committee of Beijing Ophthalmological Society, provided suggestions on eye protection during the novel coronavirus pneumonia epidemic.
[12] Review	Chinese	–	2020.02.14	Guangzhou	The delay of back-to-school time is an important measure to reduce cross infection on campus, and to protect the health of children and teenagers during the 2019 novel coronavirus outbreak. Meanwhile, remote teaching models has led to a dramatic shift in eye-use habits of children and adolescents. The potential effects on adolescent visual health cannot be ignored.
[13] Review	Chinese	–	2020.02.24	Beijing	This paper provided general guidance about precautions in ophthalmic practice in the prevention and control of the novel coronavirus pneumonia epidemic.
[14] Review	Chinese	–	2020.02.17	Beijing/Shenzhen	Some suggestions of integrated Traditional Chinese and Western Medicine are described to avoid the infection of ophthalmic medical practitioners and to effectively deal with novel coronavirus conjunctivitis during the diagnosis and treatment.
[15] Review	Chinese	–	2020.02.06	Beijing	This article briefly introduced the characteristics and identifications of SARS-CoV-2 infection, and put forward suggestions and opinions from the aspects of personal protection of ophthalmologists, control managements of ophthalmic outpatient department and ward, cleaning and disinfection of ophthalmic equipment.
[16] Review	Chinese	–	2020.02.24	Chongqing	During the prevention and control period of the epidemic of COVID-19, strict adherence to the prevention and control measures can effectively ensure the smooth implementation of the operation and the perioperative safety of medical staff and patients
[17] Case report	Chinese	30	2020.02.21	Wuhan	Three cases in 30 NCP patients with binocular conjunctivitis were found. Of them, one case visited for conjunctivitis as a first symptom and then diagnosed as NCP, and two cases visited for binocular conjunctivitis during the NCP onset. Although positive viral nucleic acid were detected in the conjunctiva sacs of 2 of other 27 NCP patients by using swabs and RT-PCR technology, no conjunctivitis occurred in these two patients.
[18] Review	Chinese	–	2020.02.21	Wenzhou	Systematic and standardized detection of viral nucleic acid and related blood factors is a necessary, fast and feasible prevention and control method in specialist ophthalmic institute during the COVID-19 epidemic.
[19] Review	Chinese	–	2020.02.17	Beijing	This paper indicated some suggestions about the management of clinical trials during novel coronavirus pneumonia outbreak including the follow-up of subjects, the treatment of epidemic serious adverse event (SAE) and the management requirements of co-sponsors, as well as the requirements and management principles for environment, subjects, examiners and inspection equipment in the process of ophthalmic clinical trials.
[20] Case report	Chinese	2	2020.03.03	Wuhan	Several COVID-19 cases with conjunctivitis or conjunctivitis as the first symptom have been observed in clinical work. This paper reports the diagnosis and treatment of one COVID-19 patient with conjunctivitis as the first symptom and one COVID-19 patient with conjunctivitis.
[21] Review	Chinese	–	2020.02.27	Xi’an	Understanding the mechanism and cell receptors of coronavirus transmission through ocular surface and the transmission characteristics of homologous coronavirus can provide some suggestions for appropriately ocular protection and identify COVID-19 coexisting with ocular signs for ophthalmologists during this epidemic disease.
[22] Review	Chinese	–	2020.02.17	Qingdao	Strengthening basic and clinical research on ocular infection caused by coronavirus should be one of the important tasks for ophthalmologists in China.
[23] Review	Chinese	–	2020.02.23	Shantou	In the absence of clinical and experimental evidence of SARS-CoV-2 in ocular infection, a retrospective literature analysis of viral pathogens that simultaneously trigger ocular lesions during the onset of epidemic diseases helps understand the methods of ocular protection in the prevention and control of the COVID-19.
[24] Cross-sectional study	Chinese	–	2020.02.27	Wenzhou	Aerosol can be produced by non-contact “air-puff” tonometer spraying, and it fluctuates with the increase of spraying times, showing a cumulative effect. The aerosol accumulation is higher in the hall with insufficient air circulation. And more aerosol can be produced without gauze mask.
[25] Review	Chinese	–	2020.03.04	Lanzhou	A review of the literature on coronavirus, especially SARS-CoV, can provide references for the prevention and control of SARS-CoV-2.
[26] Review	Chinese	–	2020.02.20	Jinan	During the SARS-CoV-2 outbreak, ophthalmologists should be alert to the potential transmission of ophthalmology-related viruses.
[27] Review	Chinese	–	2020.02.10	Shantou	This article reviews the eye performance of various types of epidemic virus infections and provides a reference for COVID-19 prevention and control.
[28] Case report	Chinese	4	2020.02.13	Wuhan	Four COVID-19 patients with conjunctivitis were mentioned in this article, and all the patients were medical staff of the hospital. One patient was positive for conjunctival sac virus nucleic acid test.
[29] Review	Chinese	–	2020.02.14	Hangzhou	According to the characteristics of previous ocular-respiratory infection viruses, ocular surface transmission may possible through the following ways: (1) infection through the nasolacrimal duct system. (2) infection through contact with eyes and nose. (3) the above two conditions exist simultaneously and cause infection.
[30] Review	Chinese	–	2020.02.18	Wuhan	This paper suggests the necessary medical protective measures for ophthalmology outpatient and ward. For the patients who were asymptomatic with the virus, there is currently a lack of effective screening methods, ophthalmologists need to be vigilant at all times.
[31] Review	Chinese	–	2020.02.18	Multi-center	Experts from multiple hospitals discussed the prevention and control system of medical staff in ophthalmic medical institutions during the epidemic of COVID-19.
[32] Review	Chinese	–	2020.03.01	Wenzhou	The authors proposed how to choose protective goggles correctly, and seven methods of anti-fog in this article.
[33] Cross-sectional study	Chinese	–	2020.03.08	Wenzhou	The Remote Dedicated Doctor Platform of Ophthalmology (RDDPO) establishes a channel for doctor-patient communication during the epidemic, which can be considered as an important way to effectively address the needs of patients for medical treatment.

### Transmission of SARS-CoV-2

For ophthalmologists, comprehensive information is needed to understand SARS-CoV-2 feature and epidemiology of the outbreak. Coronaviruses are big, enveloped, single, plus-stranded RNA viruses [34]. Seven coronavirus species are known to cause diseases in humans, among which “severe acute respiratory syndrome coronavirus” (SARS-CoV), “Middle East respiratory syndrome coronavirus”(MERS-CoV), and SARS-CoV-2 drew great attention. SARS-CoV, MERS-CoV and SARS-CoV-2 all belong to the β-CoV family and can cause fatal pneumonia. MERS-CoV carries the highest fatality rate of 34.5% [35], followed by SARS-CoV (9.6%) [36], then SARS-CoV-2 (3.70%, following data till 12th March 2020), which has a lower disease severity but higher transmission efficiency.

SARS-CoV-2 has been sequenced and was shown to be 75–80% identical to SARS-CoV and 40% identical to MERS-CoV [36]. SARS-CoV-2 shares the same host receptor with SARS-CoV, the human angiotensin-converting enzyme 2 (ACE2) receptor, suggesting a similar transmission route. The person-to-person transmission of SARS-CoV-2 can occur through respiratory droplet transmission and contact transmission. Airborne aerosol and the fecal-oral transmission route [37] remain to be further confirmed.

Theoretically, transmission through the ocular route is likely for SARS-CoV-2. First, its host receptor ACE2 has been identified on the ocular surface [38, 39]. Second, the ocular surface is an open microenvironment. Through the nasolacrimal duct, the virus may transport to the inferior meatus of the nose. Third, the ocular mucosal immune system is associated with lymphoid tissue in the nasolacrimal duct and nasal cavity [40].

There have been some studies reporting the presence of SARS-CoV or MERS-CoV in tears or conjunctival sac [41, 42], while negative results in all of included patients were also reported [43]. Several investigations have been conducted to identify whether COVID-19 can be transmitted through the ocular route. Shen and colleagues [3] performed a prospective case series study in 30 COVID-19 patients, finding SARS-CoV-2 in two conjunctival swabs of one patient. Another study by Chen et al. [1] demonstrated that out of 67 patients enrolled, SARS-CoV-2 can be detected in the conjunctival sac of three COVID-19 patients without ocular symptoms. Sun et al. found [2] that among 72 patients confirmed by laboratory diagnosis with SARS-CoV-2 RT-PCR assay, SARS-CoV-2 RNA fragments were found in ocular discharges belonging to one patient. The above studies confirmed that SARS-CoV-2 can exist in tears or the conjunctival sac, but the infection of SARS-CoV-2 through the eyes remains uncertain.

The negative results in the ocular surface may be influenced by viral concentration, sampling time lag, and diagnostic method. The time of exposure to SARS-CoV-2 infected patients is critical because of the higher viral load at the early stage of infection. Improvements in the sensitivity of molecular diagnostic methods are needed in the future. More well-designed trials with large sample sizes are required to ascertain whether the ocular route is indeed a mode of transmission.

### Precautions for SARS-CoV-2

Due to face-to-face communication with patients, frequent exposure to tears and ocular discharge, and the unavoidable use of equipment such as slit lamp, tonometer, laser etc., ophthalmologists could carry higher risks of contracting a SARS-CoV-2 infection. Ophthalmologists are recommended to take measures for mouth, nose and eyes protection when caring for patients potentially infected with SARS-CoV-2. The following recommendations on ophthalmic practice are based on the Guidelines for the Prevention and Control of Novel Coronavirus Pneumonia in Medicine, the Guideline for the Use of Medical Protective Equipment in the Prevention and Control of Novel Coronavirus Pneumonia, the list of published journal articles listed in Table 1 and clinical experience in domestic hospitals, as well as previous domestic SARS experiences and other departments such as the department of dentistry [44, 45].

#### Outpatient management

##### Before coming to hospital

- Control number of visiting patients

Reducing outpatient visitors will be critical to decrease cross-infection. Patients are asked to make an appointment before going to the hospital.

b) Make good use of online platforms

Online platforms such as the hospital’s official website or WeChat should be well utilized. Online platforms can provide notice for decreasing outpatient visits and updates on COVID-19, help patients distinguish between urgent and non-urgent ocular diseases, recommend safe and self-executing treatments for common nonurgent ocular diseases, remind patients to prepare correct personal protection before coming to the hospital, advise patients with suspicious symptoms such as fever to first visit the screening center before coming to the ophthalmic clinic, and give targeted guidance for common chronic eye diseases during this period.

c) Online ordering and delivery of prescribed medication

Both hospital and patients can benefit from submitting prescriptions online and having patient medication sent to their doorsteps via non-contact delivery.

#### Entering the hospital

- Reduce the number of accessible gateways into and out of the hospital by closing unnecessary ones

Visitors will be funneled through these gateways to allow for efficient manpower management.

**Fig. 1:**
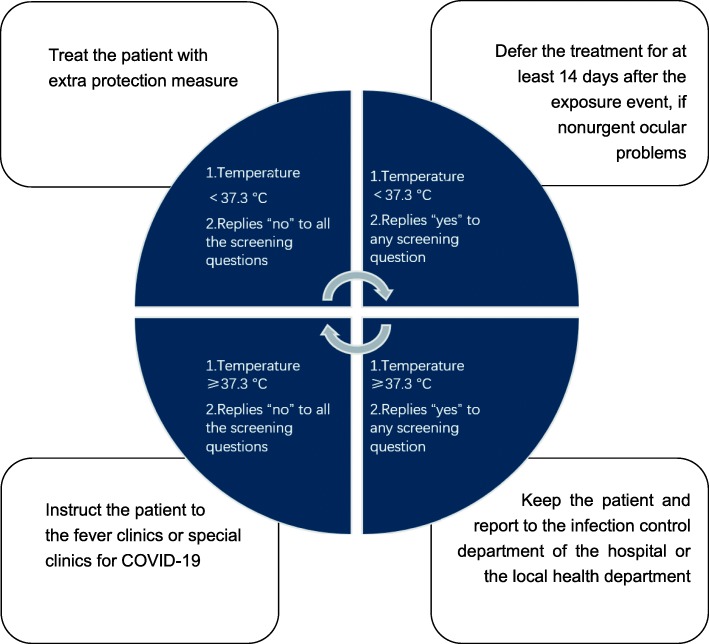
Patient screening chart

b) Set up two checkpoints in hospital entrance and treatment area entrance (Fig. 1)

Visitors should be screened through temperature measurement and symptoms before entering the hospital and treatment area.

The questionnaire includes questions about typical clinical symptoms of COVID-19, travel history to Wuhan city and other badly affected areas or countries, contact history with confirmed or suspected COVID-19 patients within the past 14 days, etc. (Fig. 1).

c) Remind patients to properly put on the mask

Every patient should wear the mask at all times in the hospital, if the self-prepared mask cannot sufficiently protect the patient, the hospital shall provide a suitable one.

d) Prepare a separate clinic for patients with symptoms of infection

Setting up a special clinic for conjunctivitis is recommended, and patients with conjunctivitis and suspicious contact/travel history should be treated for COVID-19. Patients with suspected conjunctivitis are advised to be tested more than two times for SARS-CoV-2 RNA in the conjunctival sac and tears.

##### Entering the clinic room

- Practice social distancing in the registration and waiting areas

Patients should stay at least 1.5 m apart from one another when in registration and waiting area.

b) Limit the number of people in the room

Keeping 1 doctor and 1 patient in 1 room is required except for visually impaired patients, patients with communication/mobility difficulties or parents of small children. The room should be well-ventilated. After each patient’s consultation or treatment, the used instruments such as slit lamp must be disinfected immediately.

c) Reduce outpatient examinations

Operation of many ophthalmic equipment requires close proximity, reducing outpatient examinations helps protect both doctors and patients.

Micro-aerosols can be generated when non-contact tonometry is used [46]. Air-puff ophthalmic equipment like non-contact tonometry should be avoided if unnecessary. It is advised to place the tonometer in a ventilated place, and that the measurement interval between patients should be extended. During the measurement, patients should wear a face mask.

Direct ophthalmoscope examination is not recommended, which can be replaced by slit light lens or fundus photography. Protective shields (better transparent) should be installed on slit lamps and any other equipment used which needs close doctor-patient contact. Both doctor and patient should refrain from bare face-to-face speaking during any examination.

#### Inpatient management

##### Before coming to the ward

- Control the number of scheduled surgeries

Non-emergency surgeries, such as elective cataract operations and ophthalmic plastic surgery, should be postponed. Emergency surgeries, such as endophthalmitis, eyeball rupture, macula-on rhegmatogenous retinal detachment and intraocular foreign body, can continue. Elective surgeries should still be appropriately reduced in areas where the infection is under good control.

b) Improve preoperative infection screening of inpatients

Preoperative CT examination, SARS-CoV-2 (RNA) detection, and blood routine examination are recommended. Testing of nasopharyngeal swab two or more times is recommended in suspected cases with an initially negative result [36]. If CT examination is not available for specialized hospitals or primary hospitals (lack of medical imaging department), or due to some limitations with regards to special groups (such as pregnant women), inspection of hematological indices including C-reactive protein (CRP) and serum amyloid A (SAA) are suggested as routine tests of preoperative screening for ocular surgery patients.

The infection screening results need to be checked and confirmed before surgery appointment. In general, a patient with COVID-19 is not recommended to undergo ocular surgeries unless urgent. The emergency surgeries for a COVID-19 infected patient should be arranged in a negative pressure operating room, with advance notice given to the ward and operating room. If there is no negative pressure operating room in the hospital, COVID-19 patients should go to other qualified hospitals.

##### Entering the ward

- Repeat temperature taking and query with questionnaire

Repeated temperature taking and query with questionnaire ought to be done at the ward entrance. In addition, daily temperature measurement must be a routine for all patients. The patient’s temperature and necessary examinations should not be ignored.

b) Check infection screening results of inpatients again

Infection screening results of inpatients should be checked immediately after the patient enters the ward. Repeated preoperative CT examination, SARS-CoV-2 (RNA) detection, and blood routine examination after hospital admission are also recommended by some experts.

c) Arrange a single room for every patient if possible

The necessary doctor-patient conversation can be conducted in the patient’s room. One patient is not allowed to have more than one attendant, and the attendant should be the same. Both patient and the attendant should wear the mask.

d) Reduce unnecessary inpatient examinations
e) Avoid operations under general anesthesia if possible

Operations should preferably be done under local anesthesia. The operating room should be left standing for a sufficient time between each operation after adequate disinfection measures. Operations for healthy patients will be done in a positive pressure laminar flow operating theater while for suspected or infected patients, in a negative pressure laminar flow operating theater.

#### Staff management

1. Offer relevant infection control training to the staff

The training content should include current knowledge of COVID-19, precaution measures, hand disinfection training, etc. Ophthalmologists should be able to identify a suspected case of COVID-19. Typical clinical symptoms of SARS-CoV-2 infection were onset of fever, generalized weakness, myalgia and dry cough [36, 47]. The clinical manifestations of conjunctivitis in COVID-19 patients are consistent with other viral conjunctivitis [6].

2) Report temperature and abnormal symptoms every day

Temperature taking and query with questionnaire before entering the hospital also applies to staff. Staff are advised to measure their own body temperatures twice every day and promptly report any abnormal symptoms.

3) Increase personal protection

Caps, respiratory protection, gloves, gowns, eye protection, and face shields are used for personal protection. Based on the possibility of the spread of SARS-CoV-2 infection, three-level protective measures for ophthalmic professionals are as follows (Table 2).

**Table 2:** Three-level protective measures of the ophthalmic professionals

Level of protection	Protective measures	Applicable object
Primary protection	Cap, surgical mask, and working clothes (with/without gown)	Indirect contact with patients, usher, non-operative inquiry and ward rounds.
Secondary protection	Cap, surgical mask/N95 mask, working clothes with gown, protective goggles/face shield, and disposable gloves	Direct contact with patients such as slit lamp, gonioscopy, ultrasound and other specialized examinations; puncture, injection, outpatient laser and other professional operations.
Tertiary protection	Cap, N95 mask, protective clothing with gown, protective goggles, face shield, and two pairs of disposable gloves	Contact with blood, body fluids, secretions and other spillages; specimen collection that may produce eye aerosols; internal eye surgery etc. The anesthesiologist should also adhere to this level of protection during general anesthesia operations.

Strict hand hygiene is required for every staff. It is advisable to not use a pair of latex gloves for long periods of time. Moreover, strict hand hygiene must be practiced after taking off gloves.

4) Set up inspectors and inspection group

Inspectors and inspection group will check the implementation of protective measures for members in their respective departments every day. Furthermore, they will also examine the patients’ self-protection.

#### Environmental management

1. Turn off the central air conditioning, enhance the air ventilation
2. Disinfect rooms and instruments thoroughly

Rooms and instruments should be thoroughly disinfected according to local disinfection guidelines. The elevator should be disinfected regularly. People should avoid unnecessary contact with elevator buttons and other objects when using the elevators.

#### Patient education

Patient education is crucial. It is vital to prevent nosocomial cross-infections, and thus every patient needs to pay attention to personal precautions.

Online platforms, the hospital’s official website or WeChat public platform, for example, should be well utilized before patients come to the hospital as mentioned above.

When patients are in the hospital, videos in the waiting rooms and brochures are effective approaches to teach patients updated knowledge regarding COVID-19 and personal hand hygiene, as well as remind them of proper mask wearing, and practicing social distancing.

Follow-up after discharge is easy to overlook. Despite rigorous preoperative screening, it is possible that patients in the incubation period or asymptomatic patients may be admitted for surgery. Telephone follow-up, asking about their postoperative symptoms, is very important for us to prevent unexpected virus transmission.

## Conclusions

The SARS-CoV-2 infection in China has been well controlled thanks to the large collective effort. The National Health Commission has reported that the peak of the current infection in China has passed. However, a coming nationwide resumption of work in China and developing epidemic in foreign countries are worrying; we still need to take every precaution against COVID-19.

Since 2002, coronaviruses seem to impose a continuous and enormous threat to human beings. Even as this infection dies down, we should be vigilant of future outbreaks. Ocular transmission of coronavirus remains uncertain, and more well-designed trials with large sample sizes are urgently needed.

Disclosure and sharing of knowledge are keys to controlling the outbreak. Again, we sincerely hope the Chinese experience against SARS-CoV-2 in ophthalmology can, to some extent, contribute to protecting the lives of ophthalmologists and patients worldwide.

## Data Availability

Not applicable.
